# Perspective of a pre‐symptomatic individual with an FTD‐causing MAPT gene variant

**DOI:** 10.1002/alz70858_102304

**Published:** 2025-12-26

**Authors:** Ansel Dow

**Affiliations:** ^1^ Cure MAPT FTD, DURHAM, NC, USA

## Abstract

Every living generation of my family has been affected by early‐onset genetic FTD. The disease is caused by the V337M MAPT mutation, an autosomal dominant gene variant that leads to onset of behavioral symptoms in carrier individuals between the ages of 35 and 50. My mother and five of her eight siblings have developed symptoms which have caused tremendous hardship and heartache for our family. I was tested for V337M and I learned that I am also a carrier of the variant. This information has been a heavy burden for me to bear and has guided my decisions about how I spend my healthy years and my decision not to have biological children. The knowledge that I and many of my young relatives will be affected by FTD in the future has galvanized me to become an advocate for MAPT families. Fear and social stigma often prevent at‐risk individuals from getting tested, participating in research and telling our stories. When we support each other and advocate for our needs we pave the way for development of transformative new therapies for genetic FTD.

